# Organizational citizenship behavior: adaptation and validation of the OCB scale CCOE-R

**DOI:** 10.3389/fpsyg.2024.1475011

**Published:** 2024-12-23

**Authors:** Paula C. Neves, Ana Palma-Moreira, Cláudia Andrade, Manuel Au-Yong-Oliveira

**Affiliations:** ^1^Escola Superior de Educação, Politécnico de Coimbra, Coimbra, Portugal; ^2^InED. Centro de Investigação e Inovação em Educação, Politécnico do Porto, Porto, Portugal; ^3^Faculdade de Ciências Sociais e Tecnologia, Universidade Europeia, Quinta do Bom Nome, Lisbon, Portugal; ^4^APPsyCI—Applied Psychology Research Center Capabilities & Inclusion, ISPA—Instituto Universitário, Lisbon, Portugal; ^5^GOVCOPP, University of Aveiro, Aveiro, Portugal; ^6^INESC TEC, Porto, Portugal; ^7^DEGEIT, University of Aveiro, Aveiro, Portugal

**Keywords:** organizational citizenship behavior, scale adaptation, extra-role behavior, cooperation, quantitative study

## Abstract

**Introduction:**

Organizational Citizenship Behavior has evolved as a pivotal concept in organizational behavior because of its importance on fostering the success of organizations. Despite its recognized benefits, OCB’s dimensions are not consensual in literature. The goal of this paper was to adapt and validated to be used in a broader work context an OCB scale (CCOE-R) initially developed for the Portuguese specific professional context, schools and the education sector.

**Methods:**

The sample of this study is composed of 740 participants. To validate the scale, an exploratory and a confirmatory factor analysis and scale invariance test were performed.

**Results:**

Exploratory and confirmatory factor analyses revealed a 10-item unidimensional structure, with excellent reliability indices, and goodness of fit, besides invariance for group status (managerial and non-managerial positions).

**Discussion:**

The OCB-G (Global OCB) emerges as a reliable and valid instrument that is based on a conception of OCB as a unidimensional construct. Because the items are group referenced items, it is possible to obtain a global value of OCB that represents group perceptions on OCB, allowing research to be carried out at the group/organizational level. The (CCOE-R) is an essential contribution to the study of organizational behavior, serving as a practical tool for assessing OCB-G as it plays a prominent role in organizations.

## 1 Introduction

The concept of Organizational Citizenship Behavior (OCB) is the term used in industrial and organizational psychology to describe a person’s volunteer work inside an organization that is not related to their contractual duties. Initially proposed by Smith et al. (1983), OCB builds upon earlier concepts such as Chester Barnard’s informal modes of cooperation and Katz and Kahn’s innovative behavior within open systems. Critiques by Morrison (1994) and Orr et al. (1989) prompted Organ (1977) to redefine OCB as “activities that support the social and psychological environment in which task performance occurs” (p. 95). This broader definition emphasizes behaviors that transcend routine job functions yet contribute significantly to organizational effectiveness. The research field’s significance lies in consistently identifying OCB’s positive impacts on individual and organizational performance across diverse cultures and economic sectors. However, consensus on OCB’s nature and dimensions remains not completely achieved, resulting in varied assessment instruments. While early classifications by Smith et al. (1983) categorized OCB into altruism and compliance, later expanded by Organ (1988) into five types: altruism, conscientiousness, sportsmanship, courtesy, and civic virtue. Despite the prevalence of this framework and the widely used instrument developed by Podsakoff et al. (1990), alternative structures and measures have emerged, reflecting evolving conceptualizations of OCB. Williams and Anderson (1991) introduced another dimensional structure, distinguishing OCB behaviors directed toward individuals (OCBI) from those directed toward the organization (OCBO). However, scaling OCB to group or organizational levels often involves aggregating individual data, potentially masking overall behavioral patterns (Van Mierlo et al., 2009). To address this gap for organizational-level studies, using group-referenced items like CCOE-R (Neves et al., 2014) developed a scale that ensures alignment with organizational contexts. While the scale was developed to a specific context and profession (schools and teachers) the use of the scale across diverse work environments imposes adjustments. This paper aims to adapt CCOE-R (Neves et al., 2014) for broader workplace applications, emphasizing the relevance of precise item referencing for organizational-level OCB research.

Overall, while OCB research has significantly advanced understanding of organizational dynamics, ongoing challenges in conceptualization of OCB and its’ measurement, the applicability of an instrument across contexts underscore the need for nuanced approaches ensuring that using a scale that can assess OCB in diverse organizational and workplace settings is needed for both research and practice.

The main goal of this paper was to carry out studies on adapting the CCOE-R to be used in a general work context. A secondary objective was to test this instrument in other Portuguese-speaking countries; for this purpose, the sample consisted of participants from these countries. The (CCOE-R) adapted to the organizational context (which we call OCB-G) in general could be an essential contribution to the study of organizational behavior, serving as a practical tool for assessing OCB.

## 2 Background and theoretical framework

The Organizational Citizenship Behavior concept (OCB), initially defined as “individual behavior that is discretionary, not directly or explicitly recognized by the formal reward system, and that in the aggregate promotes the effective functioning of the organization” (Organ, 1988, p. 4) has become one of the most studied topics in the field of organizational behavior. Although the designation of OCB was proposed by Smith et al. (1983), similar concepts had already been identified in the field of organizational behavior and were used as a basis to define the concept. This includes Barnard’s (1938/1968) informal modes of cooperation, which are not part of the formal bureaucratic structure, and Katz and Kahn’s (1966/1978) ‘innovative and spontaneous behavior’ within their conception of organizations as open systems. However, criticisms from Morrison (1994) regarding its discretionary nature and from Orr et al. (1989) concerning the lack of reward from superiors, led Organ (1977) to redefine the concept. He broadly defined OCB as ‘activities that support the social and psychological environment in which task performance occurs’ (p. 95). This new definition clarifies that these behaviors do not fit into routine job functions but contribute directly or indirectly to organizational effectiveness. The importance of this research field lies precisely in these aspects: the consistent identification of the positive effects of OCB on both individual and organizational performance (Podsakoff et al., 2014), in different cultures (Widarko and Anwarodin, 2022) and different economic sectors (Podsakoff et al., 2018; Rizaie et al., 2023).

Organizations where employees actively engage in OCB present good quality standards (Hadjali and Salimi, 2012); have high levels of goal achievement (Walumbwa et al., 2011), of employee work commitment (Shahab et al., 2018), knowledge sharing (Lin and Peng, 2010; Organ et al., 2005), work satisfaction and organizational loyalty and low levels of absenteeism (Chughtai and Zafar, 2006; Podsakoff et al., 2014). Overall, this means that OCB globally increases employee morale and productivity (Callea et al., 2016), as well as organizational functioning and employee benefits by creating a favorable attractive workplace and encouraging superior performance (Laski and Moosavi, 2016).

However, there is no consensus on the nature and dimensions of the construct, resulting in various instruments with different dimensional structures for assessing OCB in general. When the construct was first proposed, based on interviews with supervisors, the authors identified a set of behaviors considered to be OCB which they organized into two broad categories: altruism, that includes all behaviors with the intention of helping, and general compliance, which is less interpersonal and involves behaviors like following rules, showing up regularly and on time, and not wasting time while at work (Smith et al., 1983). Over the years, researchers identified several different conceptual frameworks that proposed different (but often very similar) types of OCB (Harvey et al., 2018).

Altogether, there are two broad types of structures in operationalization, one that focuses on the nature of behaviors and another that considers the target of these behaviors. One of the most widespread and widely used structure in research is the Organ (1988) that identified five types of OCB grouped by the nature of the behaviors namely: altruism (helping behaviors), conscientiousness (follow rules), sportsmanship (refraining from complaining), courtesy (touching base with others), and civic virtue (being involved). Later, Podsakoff et al. (1990) developed a 24-item scale to measure these behaviors. Although this five-factor structure is the most studied and the instrument by Podsakoff et al. (1990) is one of the most widely used in research, other proposed structures and instruments have emerged in recent years, as other behaviors considered to be OCB have been identified, such as Helping and Voice (Van Dyne and LePine, 1998), or protecting company resources, specific to the Chinese culture (Farh et al., 2004), or employee sustainability (Dekas et al., 2013) in line with further developments in the research of organizational citizenship behaviors that reflect the characteristics and success of the new world of work.

A different approach was proposed by Williams and Anderson (1991), who offered a dimensional structure using another criterion, suggesting that the concept of CCO integrates two wider dimensions organized according to the target of the behaviors: one that aggregates all behaviors directed at people (OCBI) and another that encompasses behaviors directed at the organization (OCBO). Despite the fact that this structure has also been confirmed in following empirical studies (Turnley, 2003), and has been widely used in literature (Henderson et al., 2020) some researchers (Hoffman et al., 2007; LePine et al., 2002; Organ, 1977) have questioned this structure as an alternative, claiming that it reflects the combination of the five dimensions initially proposed by Organ (1988). OCBI is a combination of altruism and courtesy and OCBO combines sportsmanship, civic virtue and conscientiousness.

Despite the various types and measures of OCB, there has been relatively little consistency in the specific types of OCB that researchers investigate (Harvey et al., 2018). Many types of OCB are highly correlated or share correlations, questioning the relevance of differentiating them (LePine et al., 2002).

Another issue regarding OCB measures is their level of information collection compared to the level of construct study. Most instruments collect data on OCB at the individual level, meaning the items are constructed with specific individuals as references, whether it’s oneself or someone else, such as a subordinate. However, when you want to investigate the construct at a higher level of analysis (group or organization) the most common approach is to collect individual survey responses and aggregate those, which means, researchers adopt a composition model (Chan, 1998). In other words, the data referring to the individual level are aggregated considering that they represent the OCB at the group or organizational level. Nevertheless, requesting individuals to evaluate their own behavior, or the behavior of someone, although it may highlight individual differences, it also might ignore overall patterns of behaviors within the group or organization (Van Mierlo et al., 2009). Thus, aggregating the data from individual-level items considering that they represent the average behavior of the members of the group/organization does not provide information about group perceptions on OCB because it is not a direct measure of unit-level OCBs (Ehrhart and Naumann, 2004).

In this context, it is important to consider an issue that is often ignored in group/organizational-level research: which is the referent implied in the survey items. Chan (1998) proposed a typology of composition models and emphasized the difference between self-referenced items (asking about your own behavior) and group referenced items (asking about the behavior of group elements). In this sense, if the goal is to carry out research at the organizational level it would be most coherent to clearly reference the organization and the behavior of its members as the focus of scale items.

An example of a scale that uses group-referenced items is CCOE-R (Neves et al., 2014) which makes it particularly advantageous because it enables investigation at the organizational level This scale was created to collect information that expresses the shared perceptions of the teachers regarding the levels of OCB in their schools However, as this scale was developed to be used in a specific professional context, such as schools and the education sector, which is a very particular sector, its use in different work contexts requires adaptation.

## 3 Materials and methods

### 3.1 Data collection procedure

A total of 755 individuals voluntarily participated in this study. However, only 740 were considered valid since 15 of the participants did not fulfil the essential condition for taking part in the study, which was to be working in a Portuguese-speaking country. The sampling process was non-probabilistic, for the convenience of researchers, and intentional of the snowball type (Trochim, 2000).

The questionnaire posted online on the Google Docs platform contained information about the purpose of the study. It also stated that the confidentiality of the answers would be guaranteed. After reading the informed consent form, the participants had to answer a question about their willingness to participate in the study. If they did not agree to participate in the study, they were referred to the end of the questionnaire. This study is part of an ongoing research project approved by the Ethical Committee of Polytechnic of Coimbra (Reference: 25_CEIPC_2022). The data was collected between March and May 2024.

### 3.2 Participants

This study’s sample consisted of 740 participants aged between 19 and 74 (*M* = 39.96, *SD* = 11.55). Of these participants, 433 (58.9%) were female and 307 (41.5%) were male. As for marital status, 282 (38.1%) were single, 389 (52.6%) were married or in a civil partnership, 65 (8.8%) were divorced or separated and 4 (0.5%) were widowed. Regarding the type of employment contract, 89 (12%) have an open-ended contract, 156 (21.1%) are fixed term, 421 (56.9%) are open-ended, and 74 (10%) are self-employed. About seniority in the organization, 149 (20.1%) had been in the organization for 1 year or less, 176 (23.8%) for between 1 and 3 years, 111 (15%) for between 3 and 5 years, 115 (15.5%) for between 5 and 10 years, 55 (7.4%) for between 10 and 15 years and 134 (18.1%) for more than 15 years. Among these participants, 304 (41.1%) hold a managerial position, and 436 (58.9%) do not hold any managerial position. Regarding the sector of activity, 143 (19.3%) work in the public sector, 486 (65.7%) in the private sector, 57 (7.7%) in the public/private sector and 54 (7.3%) in the social sector. The participants in this study are spread across Portugal (86.9%) and various Portuguese-speaking countries (13.1%). The distribution of participants among the various Portuguese-speaking countries is as follows: 29 (3.9%) are from Angola, 31 (4.2%) are from Cape Verde, 12 (1.6%) are from Brazil, 4 (0.5%) are from São Tomé and Príncipe, 10 (1.4%) are from Mozambique and 11 (1.5%) are from Guinea. The participants who live in Portugal are spread across all the districts of Continental Portugal and the Autonomous Regions (Table 1).

**Table 1:** Distribution of participants by district in Continental Portugal and the Autonomous Regions.

District	Frequency	Percentage
Viana do Castelo	2	0.3
Braga	19	2.6
Vila Real	3	0.4
Bragança	5	0.7
Aveiro	18	2.4
Coimbra	13	1.8
Leiria	6	0.8
Viseu	27	3.6
Guarda	4	0.5
Castelo Branco	8	1.1
Portalegre	3	0.4
Santarém	14	1.9
Évora	8	1.1
Lisboa	347	46.9
Setúbal	72	9.7
Beja	14	1.9
Faro	22	3.0
Porto	28	3.8
Funchal	10	1.4
Horta	4	0.5
Ponta Delgada	9	1.2
Angra do Heroísmo	5	0.7

### 3.3 The Organizational Citizenship Behaviors in Schools scale

The Organizational Citizenship Behaviors in Schools scale (CCOR-R) (Neves et al., 2014) was constructed in Portuguese to be used specifically in the educational context. It consists of 12 items, arranged in Likert type (classification of 1–6) ordinal scale. The items were constructed in such a way as to be organizational referenced to registers the perception each teacher has about the OCB at his/her school, and allows it, and through data aggregation to be studied at an organizational level. This instrument, adapted for teachers, had a Cronbach’s alpha of 0.90.

The questionnaire is based on OCB conception as a 2nd order latent construct with four 1st order factors (altruism, conscientiousness, civic participation and courtesy), each with four items (observable indicators) representing behaviors grouped by the nature of behavior.

This scale does not collect specific teacher information, it allows a valid index to be obtained that expresses the shared perceptions of the teachers regarding the levels of OCB of schools, making possible studies at an organizational level.

Although it was built specifically for the educational context the multi-factorial four factor structure fits the taxonomy subgroup that organizes the OCBs based on the nature of behavior and identifies itself more closely with Organ’s initial proposal (1988) for the business environment.

However, because the items were constructed specifically for the educational context, and refer to the teaching profession, their use in other contexts requires adaptation and validation. In this regard, the formulation of the items was adapted so that they could be used in a general work context. An example of this adaptation is item 1, which in the original scale was worded ‘Teachers voluntarily help new teachers’ but has been changed to ‘In my organization, employees voluntarily help new colleagues’ in the instrument we intend to validate. The response scale was changed to range from 1 ‘almost never happens’ to 5 ‘almost always happens’. Although the initial instrument consisted of 12 items, only 10 items were considered in this instrument since two of the items did not fit the general population.

The sociodemographic questionnaire consisted of the following questions: Age, gender, marital status, employment contract, seniority in the organization, sector of activity and country in which they worked. Participants working in Portugal also had an additional question about the district where they worked.

### 3.4 Data analysis procedure

After collecting the data, it was imported into SPSS Statistics 29 software (IBM Corp., Armonk, NY, USA). As the aim was to validate an instrument, the sample was randomly divided into two parts: one consisting of 304 participants, with which an exploratory factor analysis was carried out, and the other consisting of 436 participants, with which a confirmatory factor analysis was carried out. In the exploratory factor analysis, the KMO value was calculated, which should be greater than 0.70 (Sharma, 1996). This author also states that a KMO value between 0.70 and 0.80 is average, between 0.80 and 0.90 is good, and between 0.90 and 1.00 is excellent. Sharma (1996) categorizes KMO values as follows: <0.50 is unacceptable; [0.50; 0.60] is bad but still acceptable: [0.60; 0.70] is poor; [0.70; 0.80] is average; [0.80; 0.90] is good; [0.90; 1.00] is excellent. Bartlett’s test of sphericity was carried out, which indicates whether our sample comes from a multivariate population (Pestana and Gageiro, 2003). We also calculated the average variance extracted, which should be greater than 50 per cent. All items with factor weight greater than 0.50 were considered. To test internal consistency, Cronbach’s alpha was calculated, which should be higher than 0.70 (Bryman and Cramer, 2003). Regarding internal consistency, Murphy and Davidshofer (1988, p. 89) consider the following Cronbach’s alpha values: <0.60 as an unacceptable level; 0.70 as a low level; between 0.80 and 0.90 as a moderate to high level; above 0.90 as a high level.

With the other 436 participants, one confirmatory factor analyses were carried out (one factor), using AMOS Graphics for Windows software (IBM Corp., Armonk, NY, USA). The procedure followed a “model generation” logic (Jöreskog and Sörbom, 1993). Six fit indices were combined, as recommended by Hu and Bentler (1999). The fit indices calculated were as follows: Chi-squared ratio/degrees of freedom (*χ*^2^/gl); Tucker-Lewis Index (TLI); Goodness-of-fit Index (GFI); Comparative Fit Index (CFI); Root Mean Square Error of Approximation (RMSEA); Root Mean Square Residual (RMSR). The chi-square/degrees of freedom ratio (*χ*^2^/gl) must be less than 5. The CFI, GFI and TLI values must equal or exceed 0.90. As for the RMSEA, for it to be considered a good fit. Its value must be less than 0.08 (MacCallum et al., 1996). The lower the RMSR, the better the fit (Hu and Bentler, 1999). The factor weights of the items must be equal or greater than 0.50 (Hair et al., 2017). Factorial validity was assessed by calculating the standardized factor weights of the items (which should be equal to or greater than 0.50) and the individual reliability of each item (which should be equal to or greater than 0.25) (Marôco, 2021). With the data obtained from the confirmatory factor analysis, construct reliability and convergent validity were calculated (by calculating the AVE value). Construct reliability values should be greater than 0.70, and AVE values equal to or greater than 0.50 (Fornell and Larcker, 1981). However, when construct reliability value is greater than 0.70, AVE values greater than 0.40 are acceptable, indicating good convergent validity (Hair et al., 2011). The factor model measurement invariance analysis was also tested, the purpose of which is to demonstrate that the factor model proposed for each of the independent groups is invariant between the groups, i.e., that the factor weights and covariances between the factors do not differ significantly between the groups.

With the 740 participants, the sensitivity of the items was calculated, i.e., whether the items could discriminate between subjects. For this purpose, each item’s minimum, maximum, median, kurtosis and asymmetry were calculated. The items must have responses at all points, the median must not be close to one of the extremes, and the absolute values of asymmetry and kurtosis must be below 2 and 7, respectively (Finney and DiStefano, 2013). The normality of the scale was also tested.

Finally, to test the effect of the country where the participant works on the OCBs, the One-way ANOVA parametric test was carried out after checking the assumptions of normality and homogeneity of variances. Concerning normality, it should be noted that the sample tends toward normality when the group consists of more than 30 participants, according to the central limit theorem.

## 4 Results

### 4.1 Exploratory factor analysis

An exploratory factor analysis was carried out with 304 randomly selected from the 740 participants in this study. A KMO of 0.93 was obtained, which can be considered excellent (Sharma, 1996). Bartlett’s test of sphericity proved to be significant at *p* < 0.001, indicating that the sample in this study comes from a multivariate population (Pestana and Gageiro, 2003). The results indicate that the factor structure of this instrument is based on one factor instead of the four factors of the initial instrument aimed at a population of teachers. All the items have factor weights greater than 0.50 (Table 2). As for the total variance explained, the value obtained shows that this factor explains 53% of the variability in this instrument (Table 2).

**Table 2:** Results of exploratory factor analysis and internal consistency.

	Items		Factor Weights	*α*
	Portuguese	English		
1	Na minha organização os funcionários ajudam voluntariamente os novos colegas.	In my organization, employees help new colleagues voluntarily.	0.72	0.91
2	Na minha organização os funcionários oferecem-se para integrar novas equipas de trabalho.	In my organization, employees volunteer to join new work teams.	0.68	
3	Na minha organização, quando há necessidade de alguém ser substituído, o próprio toma a iniciativa de o apoiar.	In my organization, when someone needs to be replaced, they take the initiative to support them.	0.70	
4	Na minha organização os funcionários iniciam prontamente as tarefas e fazem uma gestão eficaz do tempo no desempenho das funções.	In my organization, employees start work immediately and manage their time effectively.	0.69	
5	Na minha organização, quando há necessidade de fazer qualquer alteração no horário há a preocupação de avisar com antecedência.	In my organization, when there is a need to make any changes to the schedule, there is a concern to give advance notice.	0.65	
6	Na minha organização os funcionários apresentam soluções inovadoras para melhorar a qualidade do trabalho.	In my organization, employees come up with innovative solutions to improve the quality of their work.	0.75	
7	Na minha organização os funcionários ajudam os colegas que estão mais sobrecarregados.	In my organization, employees help overloaded colleagues.	0.80	
8	Na minha organização os funcionários oferecem-se para desempenhar papéis e tarefas não obrigatórias	In my organization, employees offer to take on non-mandatory roles and tasks.	0.79	
9	Na minha organização os funcionários esforçam-se por adquirir novas competências que possam contribuir para a melhoria do seu trabalho.	In my organization, employees strive to acquire new skills that can help improve their work.	0.77	
10	Na minha organização os funcionários ajudam o chefe sempre que ele necessita e têm disponibilidade.	In my organization, employees help their managers whenever he needs it and is available.	0.71	

### 4.2 Internal consistency

As for internal consistency, this instrument has a Cronbach’s alpha value of 0.91, which can be considered a high level (Table 2).

### 4.3 Confirmatory factor analysis

A one-factor confirmatory factor analysis was carried out on the other 436 participants. As can be seen in the Table 3, the fit indices obtained in the CFA are adequate.

**Table 3:** Summary of the results of CFA, construct reliability and AVE.

Item	*λ*	*λ* ^2^	*χ*^2^/df	GFI	CFI	TLI	RMSEA	MRSR	CR	AVE
OCB1	0.66	0.37	2.41	0.97	0.98	0.97	0.057	0.033	0.90	0.46
OCB2	0.56	0.52								
OCB3	0.68	0.60								
OCB4	0.69	0.57								
OCB5	0.63	0.51								
OCB6	0.71	0.39								
OCB7	0.75	0.48								
OCB8	0.78	0.46								
OCB9	0.72	0.31								
OCB10	0.61	0.44								

All the items have factor weights greater than 0.50, which, according to Hair et al. (2017), is considered acceptable (Table 3). As the standardized factor weights (*λ*) of all the items are greater than 0.50 and their individual reliability (*λ*^2^) is greater than 0.25, factorial validity is demonstrated (Table 3) (Marôco, 2021).

Using the data obtained from the CFA, we calculated the construct reliability and convergent validity (by calculating the AVE). The instrument has a construct reliability value of 0.90, which indicates that it has good construct reliability (Table 3).

Regarding convergent validity, the AVE value is below 0.50, but as the construct reliability value is above 0.70 and the AVE value is above 0.40, convergent validity can be considered acceptable (Table 3) (Hair et al., 2011).

### 4.4 Analysis of invariance

The invariance analysis of this instrument between participants with and without a managerial position was assessed by comparing the free model (with factor weights and free factor variances/covariances) with the constructed model where the two groups’ factor weights and variances/covariances were fixed. The significance of the two models was measured using the Chi-Square test described by Marôco (2021). The constructed model, with factor weights and fixed variances/covariances in the two groups, did not show a significantly worse fit than the model with free parameters (∆*χ* 2*λ* (9) = 14.687; *p* = 0.100). It was also found that the intercepts were invariant between participants with a managerial position and those without (∆*χ* 2i (1) = 1.03; *p* = 0.309), indicating that we are dealing with a strong invariance model. The invariance of the factor model between participants with a managerial position and those without is demonstrated.

### 4.5 Sensitivity of the items and scale

As can be seen, all the items have responses at all points, and none of them has a median close to one of the extremes. However, as all the items have absolute asymmetry and kurtosis values below 2 and 7, respectively, they do not grossly violate normality, which indicates that they discriminate between subjects (Table 4).

**Table 4:** Median, minimum, maximum, asymmetry and kurtosis for each item.

	Median	Skewness	Std. Error of skewness	Kurtosis	Std. Error of kurtosis	Minimum	Maximum
OCB1	4.00	−0.705	0.090	0.257	0.179	1	5
OCB2	3.00	−0.270	0.090	−0.444	0.179	1	5
OCB3	3.00	−0.453	0.090	−0.275	0.179	1	5
OCB4	4.00	−0.643	0.090	0.485	0.179	1	5
OCB5	4.00	−0.814	0.090	0.117	0.179	1	5
OCB6	4.00	−0.438	0.090	−0.287	0.179	1	5
OCB7	3.00	−0.335	0.090	−0.507	0.179	1	5
OCB8	3.00	−0.247	0.090	−0.550	0.179	1	5
OCB9	4.00	−0.407	0.090	−0.199	0.179	1	5
OCB10	4.00	−0.568	0.090	0.136	0.179	1	5

The normality of the scale was then tested. The scale does not follow a normal distribution (*p* < 0.05). However, as it has an asymmetry of −0.43 and a kurtosis of 0.23, its absolute values of asymmetry and kurtosis are below 2 and 7, respectively, meaning it does not grossly violate normality.

### 4.6 Effect of the country on organizational citizenship behaviors

**Figure 1:**
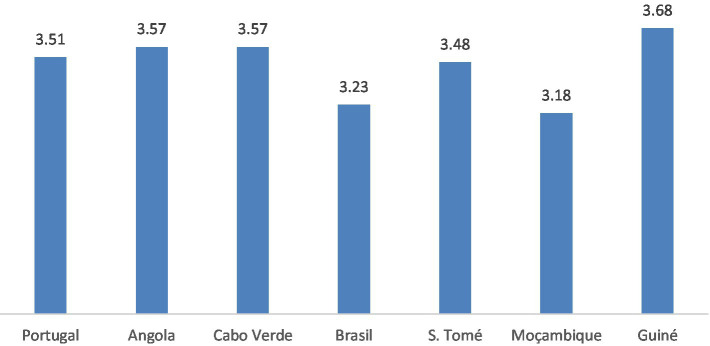
Effect of the country where the participant works on the OCBs.

Finally, the effect of the country the participant belongs to on organizational citizenship behaviors was tested. For this purpose, the One-way ANOVA parametric test was carried out after checking the respective assumptions of normality and homogeneity of variances. The assumption of normality was verified for all countries except Portugal. Still, as the Portuguese sample comprises 643 participants, according to the central limit theorem, the sample tends toward normality as it includes more than 30 participants. The assumption of homogeneity of variance was verified (*p* = 0.802). The results indicate no statistically significant differences between the different countries regarding organizational citizenship behaviors [*F*(6, 733) = 0.77, *p* = 0.592, *η*^2^*p* = 0.01]. Although the differences are not statistically significant, the country where the most organizational citizenship behaviors are practiced is Guinea, followed by Angola and Cape Verde, and the country with the lowest average is Mozambique (Figure 1).

## 5 Discussion

The aim of this study was to adapt and validate a scale of organizational citizenship behaviors for teachers, developed by Neves et al. (2014) for the Portuguese population in general. The scale was empirically tested by applying it to 740 participants.

To validate the scale, an exploratory factor analysis and a confirmatory factor analysis were carried out. The exploratory factor analysis suggested the existence of one factor. A KMO of 0.93 was obtained, which, according to Sharma (1996), can be considered excellent. All the items had factor weights equal to or greater than 0.50. The total variance explained was 53 per cent, higher than the minimum acceptable value of 50 per cent. The scale’s Cronbach’s alpha value is 0.91, which indicates a high internal consistency level, slightly higher than the alpha value of the initial instrument for teachers.

The confirmatory factor analysis confirmed the existence of one factor. The fit indices obtained are adequate. The factor weights of each item are greater than 0.50. Since each item’s standardized factor weights were higher than 0.50 and their individual reliability was higher than 0.25, this instrument has factorial validity. As for convergent validity, the AVE value is less than 0.50, the minimum acceptable value for good convergent validity (Fornell and Larcker, 1981). However, as construct reliability value of the scale is higher than 0.70, according to Hair et al. (2011), it can also be considered acceptable convergent validity. Regarding construct reliability, the scale has a value of 0.90, which can be considered good. The invariance of the factor model between participants with managerial positions and without managerial positions was demonstrated, and it was also proven that we are dealing with a model of strong invariance.

Concerning the sensitivity of the items that make up the scale, it was found that they discriminate between subjects since all the items have responses at all points, no item has a median that is close to one of the extremes, and their absolute values of asymmetry and kurtosis are less than 2 and 7, respectively (Finney and DiStefano, 2013). It was also confirmed that the scale does not grossly violate normality, thus confirming the multivariate normal distribution.

Finally, the effect of the country to which the participant belonged on organizational citizenship behaviors was tested, and no statistically significant differences were found. This indicates that the participants belonged to different Portuguese-speaking countries, which did not bias the results.

Overall, the results suggest that the OCB Scale can be considered a valid and reliable tool for researchers and practitioners to assess OCB at an organizational level.

However, the factorial structure found differs from the original scale. The original scale (CCOE-R), developed specifically for the educational context, presents a multifactorial structure, which is based on a definition of OCB as a 2nd order latent construct with 4 first order factors, each of them with 4 behavioral indicators. This structure was not confirmed with the sample of this study. The data obtained with this sample of subjects working in different work contexts, both in EFA and CFA, showed a unifactorial structure, with OCB being considered a latent construct with 10 behavioral indicators.

Although most studies consider OCB as a multidimensional construct, the operationalization of OCB as a unidimensional construct had already been stated by several researchers (Allen and Rush, 1998; Hoffman et al., 2007; LePine et al., 2002) who, based on several meta-analysis studies, found high correlations between the dimensions that in turn shared antecedent factors. In this sense, they consider that current operationalization of OCB is best viewed as indicators of a general OCB factor.

On the other hand, even studies based on a conceptualization of OCB as a multifactorial construct, in practice, are often carried out using a measure of a global OCB score obtained from the aggregation of items from the different dimensions. In other words, specific dimensions are not always differentiated and studied, but only a global OCB score is considered.

The latent construct approach may be contrasted with the aggregate approach, which is typically used in the OCB literature. In contrast to the aggregate approach, which entails averaging items taken from multidimensional OCB scales to form an overall OCB measure, the latent construct methodology involves treating existing OCB dimensions as imperfect indicators of a single construct (LePine et al., 2002). In this cases OCB is assumed as being a latent construct that integrates behaviors that are constructive and productive that workers perform resulting from their own choice, which supports colleagues and profits the organization. Thus, the items embody behavioral manifestations of positive cooperativeness at work, demonstrating a general tendency to be cooperative and helpful in organizational settings (Motowidlo, 2000; Organ, 1977). In the case of research applied to a group/or organizational level where the objective is to evaluate the shared perceptions of individuals regarding the performance of OCB, obtaining only one global score is justified.

This scale, which we call OCB-G (Global OCB), identifies behaviors considered OCB that can be recognized in work contexts in general. Although in some sectors (such as education) more relevant behaviors can be acknowledged (in the sense of contributing to organizational success) and justify evaluating categories of specific behaviors (specific dimensions), in an instrument that is intended to be suitable for all contexts, the identification of a global value of OCB seems to be more relevant, since it characterizes or defines activities that reflect the definition of OCB, i.e., activities that support the social and psychological environment in which task performance occur (Organ, 1977).

In this sense, a global concept may be more representative of a shared global perception of the group/organization’s behavioral patterns. Another characteristic of this instrument is that the focus is not on the individual but is the way the group members perceive what the “standard mode of behavior in the unit” (Ehrhart and Naumann, 2004, p.65).

The importance of OCB in the organizational context lies in its influence on the organization’s performance. Although it is individuals who perform OCB at the individual level, most actions, if considered only individually, may not make a difference to the organization’s overall performance. However, as the importance of OCB lies in their aggregation (Organ, 1988), aggregation can be done by averaging individuals’ scores or collecting data across individual groups, departments or organizations. This is what this instrument allows, as its items are group referenced items. Therefore, the value of the global score is not calculated by aggregating individually referenced responses, but rather based on responses referenced to the group. For shared perceptions of OCB between the organizational members, we believe that this rating source is more appropriate for gathering data on OCB to carry out research at the group/organizational level.

This study contributes to OCB research by providing a generic, reliable, and valid group/ organizational-level OCB measurement instrument. Additionally, it has the advantage of being potentially more accurate, since it increases its’ acceptance within organizations and groups because it measures OCB without the need to identify the individual, due to the in-measurement target.

In terms of practical implications, we can say that the use of this instrument within organizations makes it possible to capture patterns and dynamics of collective cooperation or, conversely, and because the items are group-referenced, to identify systemic patterns that may be inhibiting it, and, in this sense, to enhance interventions at the organizational level.

## 6 Conclusion, limitations and future research

The results of our study indicate that the OCB-G-Scale is a valid and reliable tool for those aiming to assess OCB in diverse organizational contexts.

Using a sample of professionals working in various organizational contexts, a unifactorial structure with OCB being considered a latent construct with 10 behavioral indicators was found. The scale proved to have very good psychometric characteristics serving as a valuable tool to assess OCB based on theoretical, practical and statistical perspectives.

Comparing the theoretical framework with the results obtained in the study it is noteworthy to consider that most OCB actions, if considered only individually, may not make a difference to the organization’s overall performance. The importance of OCB lies in their aggregation. A strong value for OCB hence appears to be an indicator of the existence of a citizenship organizational culture—one that benefits the organization’s stakeholders and. OCB may well be a calculation of the group dynamic necessary to “glue” individuals together toward a common and global goal—transcending the individuals’ functioning in their day-to-day activities. Following this line of reasoning future studies might consider how organizations/departments where employees perform OCB can account the development of team motivation, performance and organizational attractiveness. From a practical point of view having an instrument to access OCB can guide human resource managers to improve strategies to promote organizational well-being and positive functioning.

This study is not without limitations. Data collection procedure, which was non-probabilistic, intentional, and of the snowball type and the use of a self-report questionnaire, which may have biased the results should be posed as limitations of the current study.

Another limitation may be related to the fact that we did not use another instrument that measures the same construct in our data collection so that we could test the concurrent validity of the instrument that we wanted to adapt.

It should also be noted that the present study was carried out in Portuguese-speaking countries where cultural values can account to determine whether workers are more prone to perform OCB, Thus, cross-cultural studies are needed to demonstrate the robustness of the scale’s factor structure across culturally diverse groups.

Nonetheless, the (OCB-G) stands as an important contribution to the field of the study of organizational behavior, facilitating comparative studies and serving as a practical tool for assessing OCB as it continues to play a prominent role in organizational context.

## Data Availability

The raw data supporting the conclusions of this article will be made available by the authors without undue reservation.
